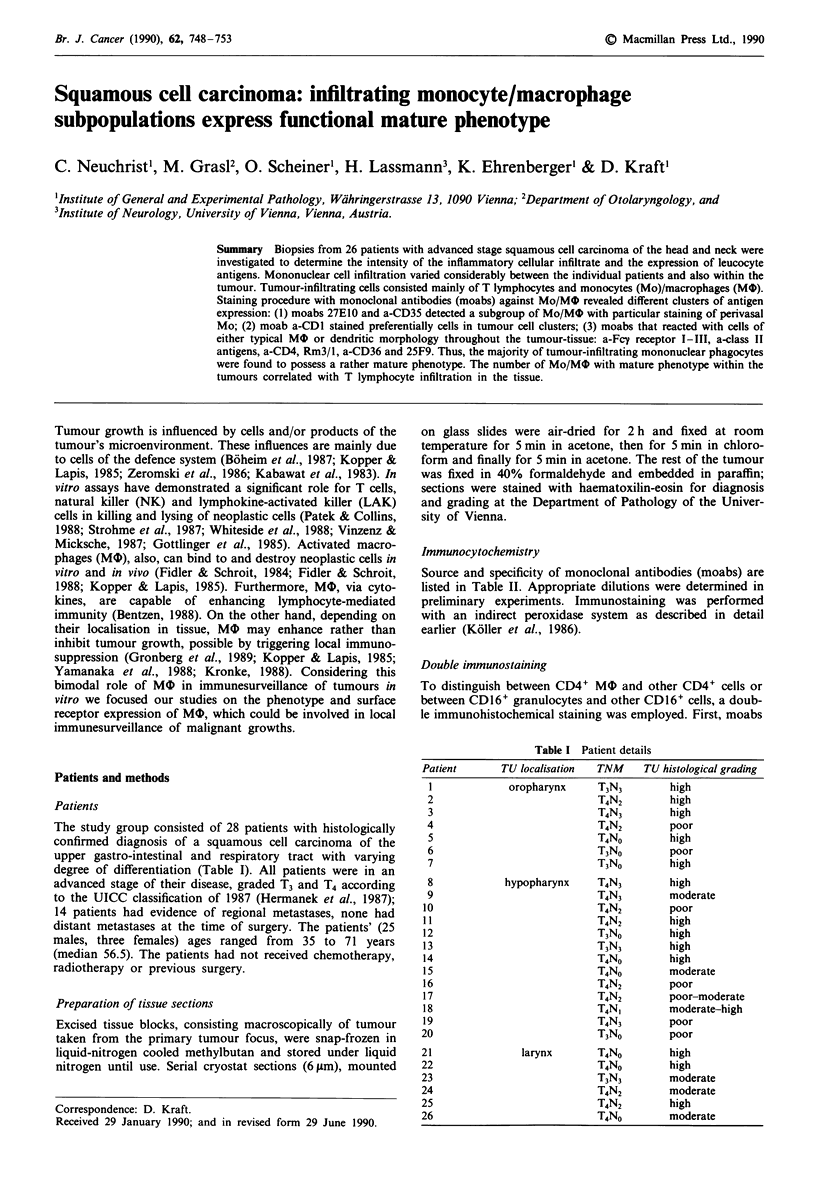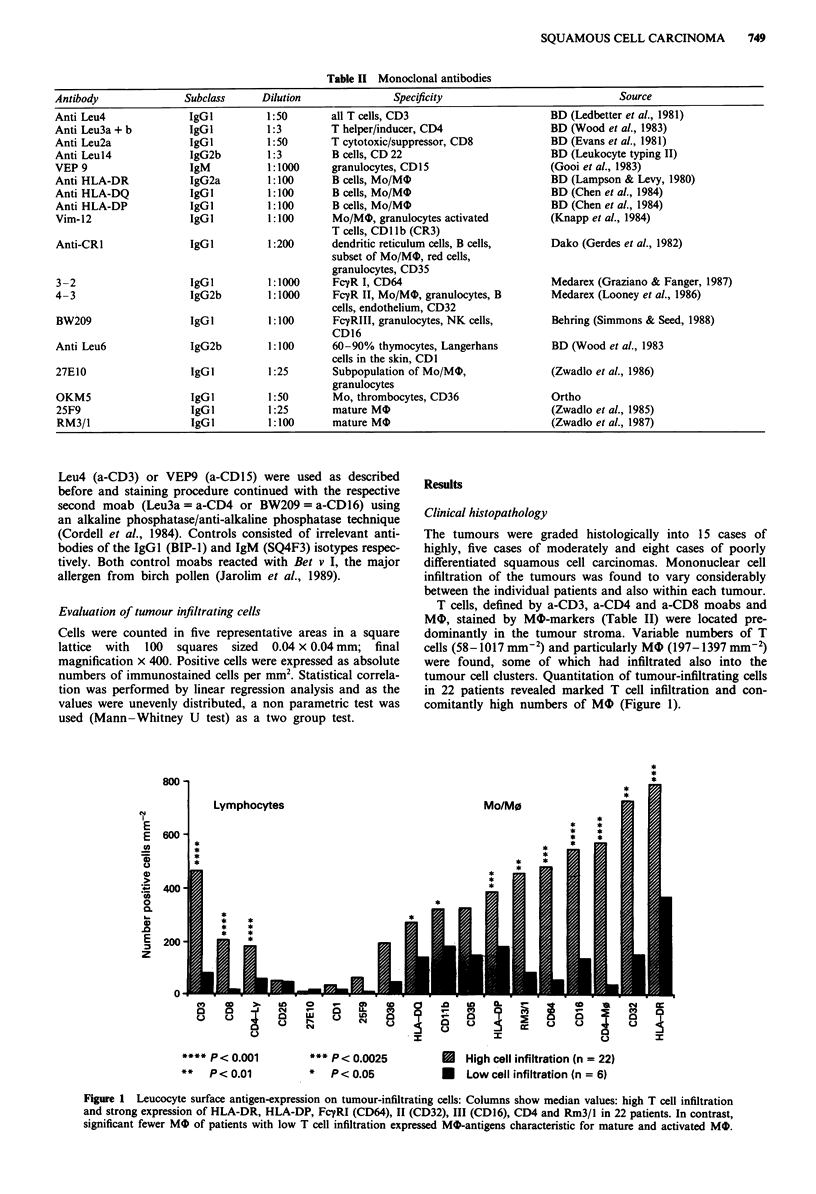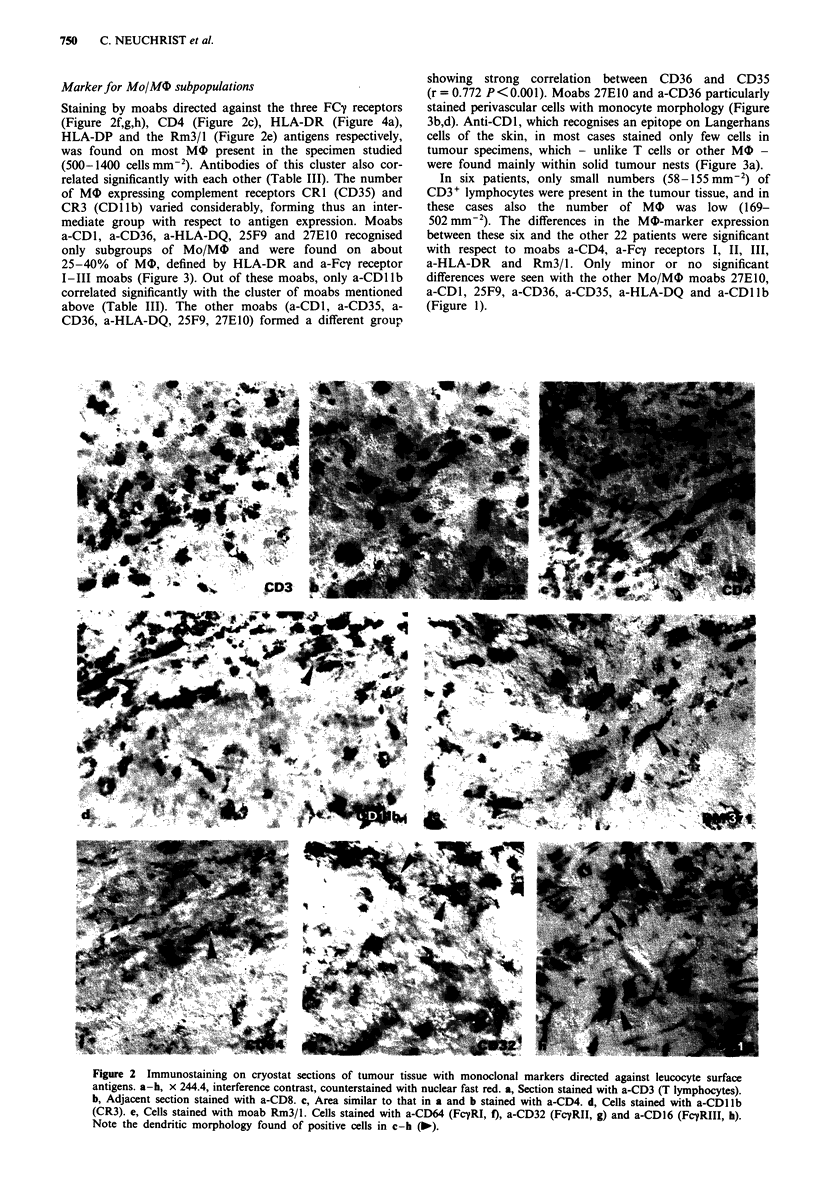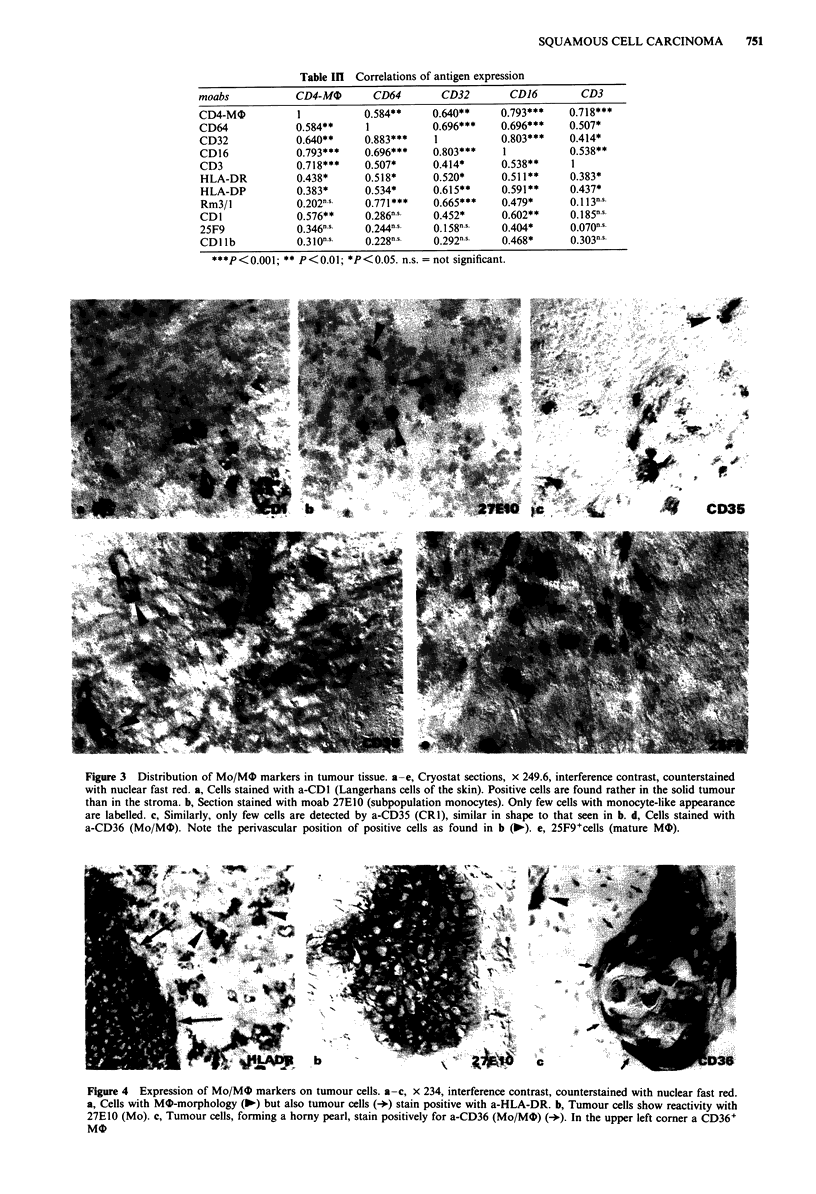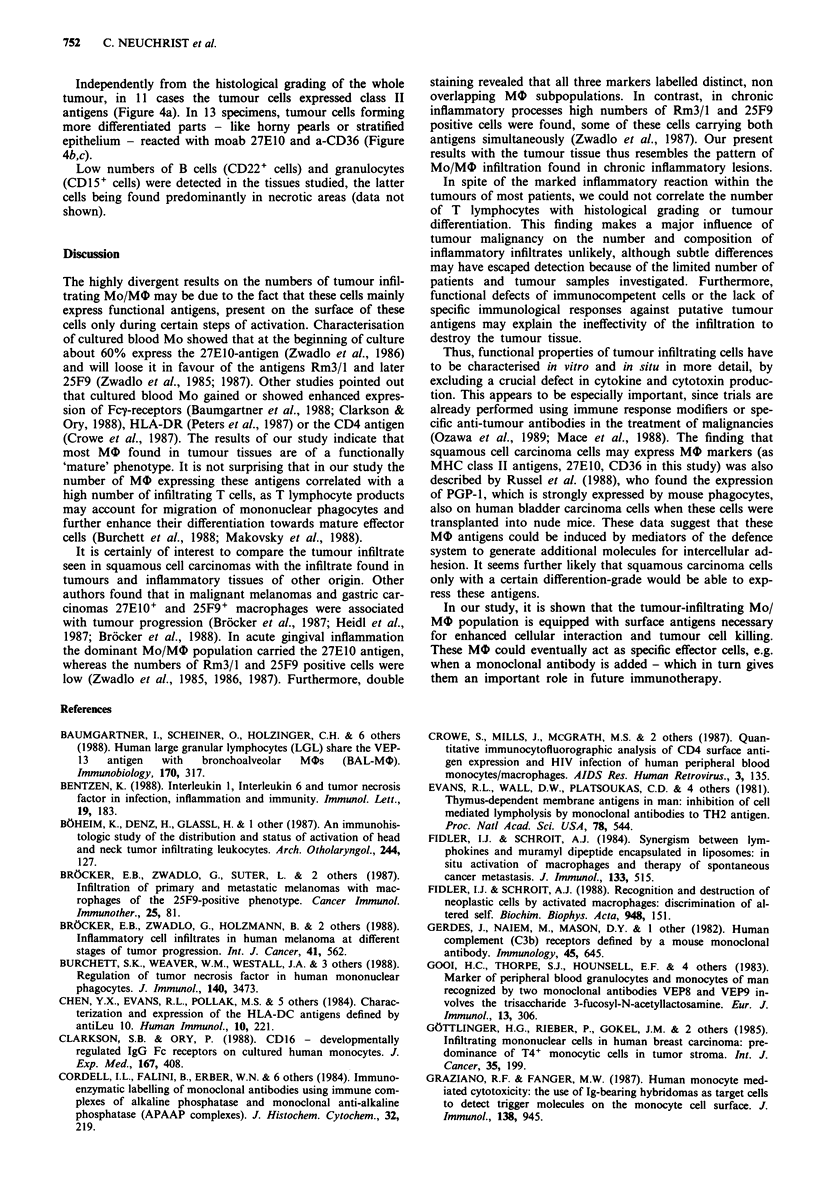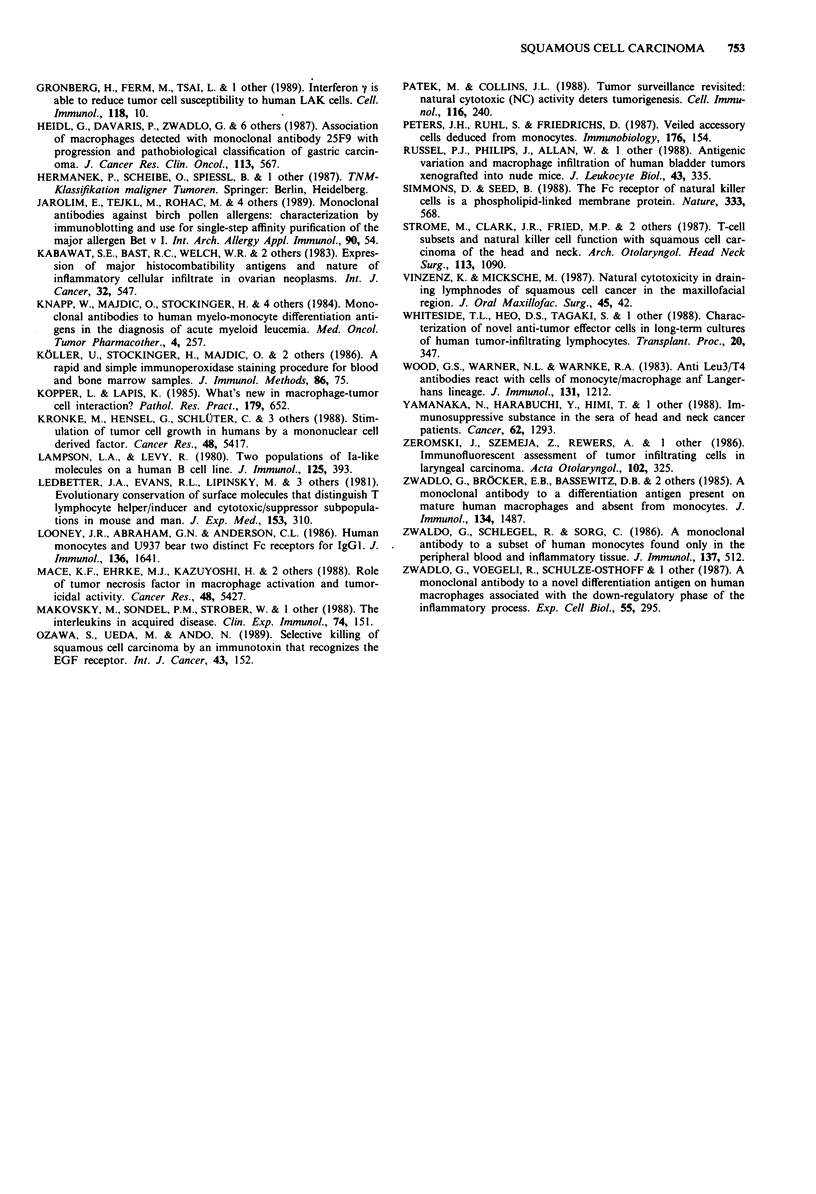# Squamous cell carcinoma: infiltrating monocyte/macrophage subpopulations express functional mature phenotype.

**DOI:** 10.1038/bjc.1990.371

**Published:** 1990-11

**Authors:** C. Neuchrist, M. Grasl, O. Scheiner, H. Lassmann, K. Ehrenberger, D. Kraft

**Affiliations:** Institute of General and Experimental Pathology, Vienna, Austria.

## Abstract

**Images:**


*Br. J. Cancer* (1990), **62**, 748–753 © Macmillan Press Ltd., 1990

### Squamous cell carcinoma: infiltrating monocyte/macrophage subpopulations express functional mature phenotype

C. Neuchrist[1], M. Grasl[2], O. Scheiner[1], H. Lassmann[3], K. Ehrenberger[1] & D. Kraft[1]

[1]*Institute of General and Experimental Pathology, Währingerstrasse 13, 1090 Vienna;* [2]*Department of Otolaryngology, and* [3]*Institute of Neurology, University of Vienna, Vienna, Austria.*

**Summary** Biopsies from 26 patients with advanced stage squamous cell carcinoma of the head and neck were investigated to determine the intensity of the inflammatory cellular infiltrate and the expression of leucocyte antigens. Mononuclear cell infiltration varied considerably between the individual patients and also within the tumour. Tumour-infiltrating cells consisted mainly of T lymphocytes and monocytes (Mo)/macrophages (MΦ). Staining procedure with monoclonal antibodies (moabs) against Mo/MΦ revealed different clusters of antigen expression: (1) moabs 27E10 and a-CD35 detected a subgroup of Mo/MΦ with particular staining of perivasal Mo; (2) moab a-CD1 stained preferentially cells in tumour cell clusters; (3) moabs that reacted with cells of either typical MΦ or dendritic morphology throughout the tumour-tissue: a-Fcγ receptor I–III, a-class II antigens, a-CD4, Rm3/1, a-CD36 and 25F9. Thus, the majority of tumour-infiltrating mononuclear phagocytes were found to possess a rather mature phenotype. The number of Mo/MΦ with mature phenotype within the tumours correlated with T lymphocyte infiltration in the tissue.

Tumour growth is influenced by cells and/or products of the tumour's microenvironment. These influences are mainly due to cells of the defence system (Böheim *et al.*, 1987; Kopper & Lapis, 1985; Zeromski *et al.*, 1986; Kabawat *et al.*, 1983). *In vitro* assays have demonstrated a significant role for T cells, natural killer (NK) and lymphokine-activated killer (LAK) cells in killing and lysing of neoplastic cells (Patek & Collins, 1988; Strohme *et al.*, 1987; Whiteside *et al.*, 1988; Vinzenz & Micksche, 1987; Gottlinger *et al.*, 1985). Activated macrophages (MΦ), also, can bind to and destroy neoplastic cells *in vitro* and *in vivo* (Fidler & Schroit, 1984; Fidler & Schroit, 1988; Kopper & Lapis, 1985). Furthermore, MΦ, via cytokines, are capable of enhancing lymphocyte-mediated immunity (Bentzen, 1988). On the other hand, depending on their localisation in tissue, MΦ may enhance rather than inhibit tumour growth, possible by triggering local immunosuppression (Gronberg *et al.*, 1989; Kopper & Lapis, 1985; Yamanaka *et al.*, 1988; Kronke, 1988). Considering this bimodal role of MΦ in immunesurveillance of tumours *in vitro* we focused our studies on the phenotype and surface receptor expression of MΦ, which could be involved in local immunesurveillance of malignant growths.

### Patients and methods

### *Patients*

The study group consisted of 28 patients with histologically confirmed diagnosis of a squamous cell carcinoma of the upper gastro-intestinal and respiratory tract with varying degree of differentiation (Table I). All patients were in an advanced stage of their disease, graded $T_3$ and $T_4$ according to the UICC classification of 1987 (Hermanek *et al.*, 1987); 14 patients had evidence of regional metastases, none had distant metastases at the time of surgery. The patients' (25 males, three females) ages ranged from 35 to 71 years (median 56.5). The patients had not received chemotherapy, radiotherapy or previous surgery.

### *Preparation of tissue sections*

Excised tissue blocks, consisting macroscopically of tumour taken from the primary tumour focus, were snap-frozen in liquid-nitrogen cooled methylbutan and stored under liquid nitrogen until use. Serial cryostat sections (6 μm), mounted on glass slides were air-dried for 2 h and fixed at room temperature for 5 min in acetone, then for 5 min in chloroform and finally for 5 min in acetone. The rest of the tumour was fixed in 40% formaldehyde and embedded in paraffin; sections were stained with haematoxilin-eosin for diagnosis and grading at the Department of Pathology of the University of Vienna.

### *Immunocytochemistry*

Source and specificity of monoclonal antibodies (moabs) are listed in Table II. Appropriate dilutions were determined in preliminary experiments. Immunostaining was performed with an indirect peroxidase system as described in detail earlier (Köller *et al.*, 1986).

### *Double immunostaining*

To distinguish between $CD4^+$ MΦ and other $CD4^+$ cells or between $CD16^+$ granulocytes and other $CD16^+$ cells, a double immunohistochemical staining was employed. First, moabs

**Table I** Patient details

| *Patient* | *TU localisation* | *TNM* | *TU histological grading* |
|---|---|---|---|
| 1 | oropharynx | $T_3N_3$ | high |
| 2 | | $T_4N_2$ | high |
| 3 | | $T_4N_3$ | high |
| 4 | | $T_4N_2$ | poor |
| 5 | | $T_4N_0$ | high |
| 6 | | $T_3N_0$ | poor |
| 7 | | $T_3N_0$ | high |
| 8 | hypopharynx | $T_4N_3$ | high |
| 9 | | $T_4N_3$ | moderate |
| 10 | | $T_4N_2$ | poor |
| 11 | | $T_4N_2$ | high |
| 12 | | $T_3N_0$ | high |
| 13 | | $T_3N_3$ | high |
| 14 | | $T_4N_0$ | high |
| 15 | | $T_4N_0$ | moderate |
| 16 | | $T_4N_2$ | poor |
| 17 | | $T_4N_2$ | poor–moderate |
| 18 | | $T_4N_1$ | moderate–high |
| 19 | | $T_4N_3$ | poor |
| 20 | | $T_3N_0$ | poor |
| 21 | larynx | $T_4N_0$ | high |
| 22 | | $T_4N_0$ | high |
| 23 | | $T_3N_3$ | moderate |
| 24 | | $T_4N_2$ | moderate |
| 25 | | $T_4N_2$ | high |
| 26 | | $T_4N_0$ | moderate |

Correspondence: D. Kraft.
Received 29 January 1990; and in revised form 29 June 1990.

**Table II** Monoclonal antibodies

| *Antibody* | *Subclass* | *Dilution* | *Specificity* | *Source* |
|---|---|---|---|---|
| Anti Leu4 | IgG1 | 1:50 | all T cells, CD3 | BD (Ledbetter *et al.*, 1981) |
| Anti Leu3a + b | IgG1 | 1:3 | T helper/inducer, CD4 | BD (Wood *et al.*, 1983) |
| Anti Leu2a | IgG1 | 1:50 | T cytotoxic/suppressor, CD8 | BD (Evans *et al.*, 1981) |
| Anti Leu14 | IgG2b | 1:3 | B cells, CD 22 | BD (Leukocyte typing II) |
| VEP 9 | IgM | 1:1000 | granulocytes, CD15 | (Gooi *et al.*, 1983) |
| Anti HLA-DR | IgG2a | 1:100 | B cells, Mo/MΦ | BD (Lampson & Levy, 1980) |
| Anti HLA-DQ | IgG1 | 1:100 | B cells, Mo/MΦ | BD (Chen *et al.*, 1984) |
| Anti HLA-DP | IgG1 | 1:100 | B cells, Mo/MΦ | BD (Chen *et al.*, 1984) |
| Vim-12 | IgG1 | 1:100 | Mo/MΦ, granulocytes activated T cells, CD11b (CR3) | (Knapp *et al.*, 1984) |
| Anti-CR1 | IgG1 | 1:200 | dendritic reticulum cells, B cells, subset of Mo/MΦ, red cells, granulocytes, CD35 | Dako (Gerdes *et al.*, 1982) |
| 3-2 | IgG1 | 1:1000 | FcγR I, CD64 | Medarex (Graziano & Fanger, 1987) |
| 4-3 | IgG2b | 1:1000 | FcγR II, Mo/MΦ, granulocytes, B cells, endothelium, CD32 | Medarex (Looney *et al.*, 1986) |
| BW209 | IgG1 | 1:100 | FcγRIII, granulocytes, NK cells, CD16 | Behring (Simmons & Seed, 1988) |
| Anti Leu6 | IgG2b | 1:100 | 60-90% thymocytes, Langerhans cells in the skin, CD1 | BD (Wood *et al.*, 1983 |
| 27E10 | IgG1 | 1:25 | Subpopulation of Mo/MΦ, granulocytes | (Zwadlo *et al.*, 1986) |
| OKM5 | IgG1 | 1:50 | Mo, thrombocytes, CD36 | Ortho |
| 25F9 | IgG1 | 1:25 | mature MΦ | (Zwadlo *et al.*, 1985) |
| RM3/1 | IgG1 | 1:100 | mature MΦ | (Zwadlo *et al.*, 1987) |

Leu4 (a-CD3) or VEP9 (a-CD15) were used as described before and staining procedure continued with the respective second moab (Leu3a = a-CD4 or BW209 = a-CD16) using an alkaline phosphatase/anti-alkaline phosphatase technique (Cordell *et al.*, 1984). Controls consisted of irrelevant antibodies of the IgG1 (BIP-1) and IgM (SQ4F3) isotypes respectively. Both control moabs reacted with *Bet v* I, the major allergen from birch pollen (Jarolim *et al.*, 1989).

### *Evaluation of tumour infiltrating cells*

Cells were counted in five representative areas in a square lattice with 100 squares sized 0.04 × 0.04 mm; final magnification × 400. Positive cells were expressed as absolute numbers of immunostained cells per $mm^2$. Statistical correlation was performed by linear regression analysis and as the values were unevenly distributed, a non parametric test was used (Mann-Whitney U test) as a two group test.

### Results

### *Clinical histopathology*

The tumours were graded histologically into 15 cases of highly, five cases of moderately and eight cases of poorly differentiated squamous cell carcinomas. Mononuclear cell infiltration of the tumours was found to vary considerably between the individual patients and also within each tumour.

T cells, defined by a-CD3, a-CD4 and a-CD8 moabs and MΦ, stained by MΦ-markers (Table II) were located predominantly in the tumour stroma. Variable numbers of T cells (58-1017 $mm^{-2}$) and particularly MΦ (197-1397 $mm^{-2}$) were found, some of which had infiltrated also into the tumour cell clusters. Quantitation of tumour-infiltrating cells in 22 patients revealed marked T cell infiltration and concomitantly high numbers of MΦ (Figure 1).

**Figure 1** Leucocyte surface antigen-expression on tumour-infiltrating cells: Columns show median values: high T cell infiltration and strong expression of HLA-DR, HLA-DP, FcγRI (CD64), II (CD32), III (CD16), CD4 and Rm3/1 in 22 patients. In contrast, significant fewer MΦ of patients with low T cell infiltration expressed MΦ-antigens characteristic for mature and activated MΦ.

*Marker for Mo/MΦ subpopulations*

Staining by moabs directed against the three FCγ receptors (Figure 2f,g,h), CD4 (Figure 2c), HLA-DR (Figure 4a), HLA-DP and the Rm3/1 (Figure 2e) antigens respectively, was found on most MΦ present in the specimen studied (500–1400 cells $mm^{-2}$). Antibodies of this cluster also correlated significantly with each other (Table III). The number of MΦ expressing complement receptors CR1 (CD35) and CR3 (CD11b) varied considerably, forming thus an intermediate group with respect to antigen expression. Moabs a-CD1, a-CD36, a-HLA-DQ, 25F9 and 27E10 recognised only subgroups of Mo/MΦ and were found on about 25–40% of MΦ, defined by HLA-DR and a-Fcγ receptor I–III moabs (Figure 3). Out of these moabs, only a-CD11b correlated significantly with the cluster of moabs mentioned above (Table III). The other moabs (a-CD1, a-CD35, a-CD36, a-HLA-DQ, 25F9, 27E10) formed a different group showing strong correlation between CD36 and CD35 ($r = 0.772$ $P < 0.001$). Moabs 27E10 and a-CD36 particularly stained perivascular cells with monocyte morphology (Figure 3b,d). Anti-CD1, which recognises an epitope on Langerhans cells of the skin, in most cases stained only few cells in tumour specimens, which – unlike T cells or other MΦ – were found mainly within solid tumour nests (Figure 3a).

In six patients, only small numbers (58–155 $mm^{-2}$) of $CD3^{+}$ lymphocytes were present in the tumour tissue, and in these cases also the number of MΦ was low (169–502 $mm^{-2}$). The differences in the MΦ-marker expression between these six and the other 22 patients were significant with respect to moabs a-CD4, a-Fcγ receptors I, II, III, a-HLA-DR and Rm3/1. Only minor or no significant differences were seen with the other Mo/MΦ moabs 27E10, a-CD1, 25F9, a-CD36, a-CD35, a-HLA-DQ and a-CD11b (Figure 1).

**Figure 2** Immunostaining on cryostat sections of tumour tissue with monoclonal markers directed against leucocyte surface antigens. **a**–**h**, × 244.4, interference contrast, counterstained with nuclear fast red. **a**, Section stained with a-CD3 (T lymphocytes). **b**, Adjacent section stained with a-CD8. **c**, Area similar to that in **a** and **b** stained with a-CD4. **d**, Cells stained with a-CD11b (CR3). **e**, Cells stained with moab Rm3/1. Cells stained with a-CD64 (FcγRI, **f**), a-CD32 (FcγRII, **g**) and a-CD16 (FcγRIII, **h**). Note the dendritic morphology found of positive cells in **c**–**h** (▶).

**Table III** Correlations of antigen expression

| *moabs* | *CD4-MΦ* | *CD64* | *CD32* | *CD16* | *CD3* |
|---|---|---|---|---|---|
| CD4-MΦ | 1 | 0.584** | 0.640** | 0.793*** | 0.718*** |
| CD64 | 0.584** | 1 | 0.696*** | 0.696*** | 0.507* |
| CD32 | 0.640** | 0.883*** | 1 | 0.803*** | 0.414* |
| CD16 | 0.793*** | 0.696*** | 0.803*** | 1 | 0.538** |
| CD3 | 0.718*** | 0.507* | 0.414* | 0.538** | 1 |
| HLA-DR | 0.438* | 0.518* | 0.520* | 0.511** | 0.383* |
| HLA-DP | 0.383* | 0.534* | 0.615** | 0.591** | 0.437* |
| Rm3/1 | 0.202$^{n.s.}$ | 0.771*** | 0.665*** | 0.479* | 0.113$^{n.s.}$ |
| CD1 | 0.576** | 0.286$^{n.s.}$ | 0.452* | 0.602** | 0.185$^{n.s.}$ |
| 25F9 | 0.346$^{n.s.}$ | 0.244$^{n.s.}$ | 0.158$^{n.s.}$ | 0.404* | 0.070$^{n.s.}$ |
| CD11b | 0.310$^{n.s.}$ | 0.228$^{n.s.}$ | 0.292$^{n.s.}$ | 0.468* | 0.303$^{n.s.}$ |

***$P<0.001$; ** $P<0.01$; *$P<0.05$. n.s. = not significant.

**Figure 3** Distribution of Mo/MΦ markers in tumour tissue. **a**–**e**, Cryostat sections, × 249.6, interference contrast, counterstained with nuclear fast red. **a**, Cells stained with a-CD1 (Langerhans cells of the skin). Positive cells are found rather in the solid tumour than in the stroma. **b**, Section stained with moab 27E10 (subpopulation monocytes). Only few cells with monocyte-like appearance are labelled. **c**, Similarly, only few cells are detected by a-CD35 (CR1), similar in shape to that seen in **b**. **d**, Cells stained with a-CD36 (Mo/MΦ). Note the perivascular position of positive cells as found in **b** (►). **e**, 25F9$^+$cells (mature MΦ).

**Figure 4** Expression of Mo/MΦ markers on tumour cells. **a**–**c**, × 234, interference contrast, counterstained with nuclear fast red. **a**, Cells with MΦ-morphology (►) but also tumour cells (→) stain positive with a-HLA-DR. **b**, Tumour cells show reactivity with 27E10 (Mo). **c**, Tumour cells, forming a horny pearl, stain positively for a-CD36 (Mo/MΦ) (→). In the upper left corner a CD36$^+$ MΦ

Independently from the histological grading of the whole tumour, in 11 cases the tumour cells expressed class II antigens (Figure 4a). In 13 specimens, tumour cells forming more differentiated parts – like horny pearls or stratified epithelium – reacted with moab 27E10 and a-CD36 (Figure 4*b,c*).

Low numbers of B cells (CD22$^+$ cells) and granulocytes (CD15$^+$ cells) were detected in the tissues studied, the latter cells being found predominantly in necrotic areas (data not shown).

### Discussion

The highly divergent results on the numbers of tumour infiltrating Mo/MΦ may be due to the fact that these cells mainly express functional antigens, present on the surface of these cells only during certain steps of activation. Characterisation of cultured blood Mo showed that at the beginning of culture about 60% express the 27E10-antigen (Zwadlo *et al.*, 1986) and will loose it in favour of the antigens Rm3/1 and later 25F9 (Zwadlo *et al.*, 1985; 1987). Other studies pointed out that cultured blood Mo gained or showed enhanced expression of Fcγ-receptors (Baumgartner *et al.*, 1988; Clarkson & Ory, 1988), HLA-DR (Peters *et al.*, 1987) or the CD4 antigen (Crowe *et al.*, 1987). The results of our study indicate that most MΦ found in tumour tissues are of a functionally 'mature' phenotype. It is not surprising that in our study the number of MΦ expressing these antigens correlated with a high number of infiltrating T cells, as T lymphocyte products may account for migration of mononuclear phagocytes and further enhance their differentiation towards mature effector cells (Burchett *et al.*, 1988; Makovsky *et al.*, 1988).

It is certainly of interest to compare the tumour infiltrate seen in squamous cell carcinomas with the infiltrate found in tumours and inflammatory tissues of other origin. Other authors found that in malignant melanomas and gastric carcinomas 27E10$^+$ and 25F9$^+$ macrophages were associated with tumour progression (Bröcker *et al.*, 1987; Heidl *et al.*, 1987; Bröcker *et al.*, 1988). In acute gingival inflammation the dominant Mo/MΦ population carried the 27E10 antigen, whereas the numbers of Rm3/1 and 25F9 positive cells were low (Zwadlo *et al.*, 1985, 1986, 1987). Furthermore, double staining revealed that all three markers labelled distinct, non overlapping MΦ subpopulations. In contrast, in chronic inflammatory processes high numbers of Rm3/1 and 25F9 positive cells were found, some of these cells carrying both antigens simultaneously (Zwadlo *et al.*, 1987). Our present results with the tumour tissue thus resembles the pattern of Mo/MΦ infiltration found in chronic inflammatory lesions.

In spite of the marked inflammatory reaction within the tumours of most patients, we could not correlate the number of T lymphocytes with histological grading or tumour differentiation. This finding makes a major influence of tumour malignancy on the number and composition of inflammatory infiltrates unlikely, although subtle differences may have escaped detection because of the limited number of patients and tumour samples investigated. Furthermore, functional defects of immunocompetent cells or the lack of specific immunological responses against putative tumour antigens may explain the ineffectivity of the infiltration to destroy the tumour tissue.

Thus, functional properties of tumour infiltrating cells have to be characterised *in vitro* and *in situ* in more detail, by excluding a crucial defect in cytokine and cytotoxin production. This appears to be especially important, since trials are already performed using immune response modifiers or specific anti-tumour antibodies in the treatment of malignancies (Ozawa *et al.*, 1989; Mace *et al.*, 1988). The finding that squamous cell carcinoma cells may express MΦ markers (as MHC class II antigens, 27E10, CD36 in this study) was also described by Russel *et al.* (1988), who found the expression of PGP-1, which is strongly expressed by mouse phagocytes, also on human bladder carcinoma cells when these cells were transplanted into nude mice. These data suggest that these MΦ antigens could be induced by mediators of the defence system to generate additional molecules for intercellular adhesion. It seems further likely that squamous carcinoma cells only with a certain differention-grade would be able to express these antigens.

In our study, it is shown that the tumour-infiltrating Mo/MΦ population is equipped with surface antigens necessary for enhanced cellular interaction and tumour cell killing. These MΦ could eventually act as specific effector cells, e.g. when a monoclonal antibody is added – which in turn gives them an important role in future immunotherapy.

### References

BAUMGARTNER, I., SCHEINER, O., HOLZINGER, C.H. & 6 others (1988). Human large granular lymphocytes (LGL) share the VEP-13 antigen with bronchoalveolar MΦs (BAL-MΦ). *Immunobiology*, **170**, 317.

BENTZEN, K. (1988). Interleukin 1, Interleukin 6 and tumor necrosis factor in infection, inflammation and immunity. *Immunol. Lett.*, **19**, 183.

BÖHEIM, K., DENZ, H., GLASSL, H. & 1 other (1987). An immunohistologic study of the distribution and status of activation of head and neck tumor infiltrating leukocytes. *Arch. Otholaryngol.*, **244**, 127.

BRÖCKER, E.B., ZWADLO, G., SUTER, L. & 2 others (1987). Infiltration of primary and metastatic melanomas with macrophages of the 25F9-positive phenotype. *Cancer Immunol. Immunother.*, **25**, 81.

BRÖCKER, E.B., ZWADLO, G., HOLZMANN, B. & 2 others (1988). Inflammatory cell infiltrates in human melanoma at different stages of tumor progression. *Int. J. Cancer*, **41**, 562.

BURCHETT, S.K., WEAVER, W.M., WESTALL, J.A. & 3 others (1988). Regulation of tumor necrosis factor in human mononuclear phagocytes. *J. Immunol.*, **140**, 3473.

CHEN, Y.X., EVANS, R.L., POLLAK, M.S. & 5 others (1984). Characterization and expression of the HLA-DC antigens defined by antiLeu 10. *Human Immunol.*, **10**, 221.

CLARKSON, S.B. & ORY, P. (1988). CD16 – developmentally regulated IgG Fc receptors on cultured human monocytes. *J. Exp. Med.*, **167**, 408.

CORDELL, I.L., FALINI, B., ERBER, W.N. & 6 others (1984). Immunoenzymatic labelling of monoclonal antibodies using immune complexes of alkaline phosphatase and monoclonal anti-alkaline phosphatase (APAAP complexes). *J. Histochem. Cytochem.*, **32**, 219.

CROWE, S., MILLS, J., MCGRATH, M.S. & 2 others (1987). Quantitative immunocytofluorographic analysis of CD4 surface antigen expression and HIV infection of human peripheral blood monocytes/macrophages. *AIDS Res. Human Retrovirus.*, **3**, 135.

EVANS, R.L., WALL, D.W., PLATSOUKAS, C.D. & 4 others (1981). Thymus-dependent membrane antigens in man: inhibition of cell mediated lympholysis by monoclonal antibodies to TH2 antigen. *Proc. Natl Acad. Sci. USA*, **78**, 544.

FIDLER, I.J. & SCHROIT, A.J. (1984). Synergism between lymphokines and muramyl dipeptide encapsulated in liposomes: in situ activation of macrophages and therapy of spontaneous cancer metastasis. *J. Immunol.*, **133**, 515.

FIDLER, I.J. & SCHROIT, A.J. (1988). Recognition and destruction of neoplastic cells by activated macrophages: discrimination of altered self. *Biochim. Biophys. Acta*, **948**, 151.

GERDES, J., NAIEM, M., MASON, D.Y. & 1 other (1982). Human complement (C3b) receptors defined by a mouse monoclonal antibody. *Immunology*, **45**, 645.

GOOI, H.C., THORPE, S.J., HOUNSELL, E.F. & 4 others (1983). Marker of peripheral blood granulocytes and monocytes of man recognized by two monoclonal antibodies VEP8 and VEP9 involves the trisaccharide 3-fucosyl-N-acetyllactosamine. *Eur. J. Immunol.*, **13**, 306.

GÖTTLINGER, H.G., RIEBER, P., GOKEL, J.M. & 2 others (1985). Infiltrating mononuclear cells in human breast carcinoma: predominance of T4$^+$ monocytic cells in tumor stroma. *Int. J. Cancer*, **35**, 199.

GRAZIANO, R.F. & FANGER, M.W. (1987). Human monocyte mediated cytotoxicity: the use of Ig-bearing hybridomas as target cells to detect trigger molecules on the monocyte cell surface. *J. Immunol.*, **138**, 945.

GRONBERG, H., FERM, M., TSAI, L. & 1 other (1989). Interferon γ is able to reduce tumor cell susceptibility to human LAK cells. *Cell. Immunol.*, **118**, 10.

HEIDL, G., DAVARIS, P., ZWADLO, G. & 6 others (1987). Association of macrophages detected with monoclonal antibody 25F9 with progression and pathobiological classification of gastric carcinoma. *J. Cancer Res. Clin. Oncol.*, **113**, 567.

HERMANEK, P., SCHEIBE, O., SPIESSL, B. & 1 other (1987). *TNM-Klassifikation maligner Tumoren*. Springer: Berlin, Heidelberg.

JAROLIM, E., TEJKL, M., ROHAC, M. & 4 others (1989). Monoclonal antibodies against birch pollen allergens: characterization by immunoblotting and use for single-step affinity purification of the major allergen Bet v I. *Int. Arch. Allergy Appl. Immunol.*, **90**, 54.

KABAWAT, S.E., BAST, R.C., WELCH, W.R. & 2 others (1983). Expression of major histocombatibility antigens and nature of inflammatory cellular infiltrate in ovarian neoplasms. *Int. J. Cancer*, **32**, 547.

KNAPP, W., MAJDIC, O., STOCKINGER, H. & 4 others (1984). Monoclonal antibodies to human myelo-monocyte differentiation antigens in the diagnosis of acute myeloid leucemia. *Med. Oncol. Tumor Pharmacother.*, **4**, 257.

KÖLLER, U., STOCKINGER, H., MAJDIC, O. & 2 others (1986). A rapid and simple immunoperoxidase staining procedure for blood and bone marrow samples. *J. Immunol. Methods*, **86**, 75.

KOPPER, L. & LAPIS, K. (1985). What's new in macrophage-tumor cell interaction? *Pathol. Res. Pract.*, **179**, 652.

KRONKE, M., HENSEL, G., SCHLÜTER, C. & 3 others (1988). Stimulation of tumor cell growth in humans by a mononuclear cell derived factor. *Cancer Res.*, **48**, 5417.

LAMPSON, L.A., & LEVY, R. (1980). Two populations of Ia-like molecules on a human B cell line. *J. Immunol.*, **125**, 393.

LEDBETTER, J.A., EVANS, R.L., LIPINSKY, M. & 3 others (1981). Evolutionary conservation of surface molecules that distinguish T lymphocyte helper/inducer and cytotoxic/suppressor subpopulations in mouse and man. *J. Exp. Med.*, **153**, 310.

LOONEY, J.R., ABRAHAM, G.N. & ANDERSON, C.L. (1986). Human monocytes and U937 bear two distinct Fc receptors for IgG1. *J. Immunol.*, **136**, 1641.

MACE, K.F., EHRKE, M.J., KAZUYOSHI, H. & 2 others (1988). Role of tumor necrosis factor in macrophage activation and tumoricidal activity. *Cancer Res.*, **48**, 5427.

MAKOVSKY, M., SONDEL, P.M., STROBER, W. & 1 other (1988). The interleukins in acquired disease. *Clin. Exp. Immunol.*, **74**, 151.

OZAWA, S., UEDA, M. & ANDO, N. (1989). Selective killing of squamous cell carcinoma by an immunotoxin that recognizes the EGF receptor. *Int. J. Cancer*, **43**, 152.

PATEK, M. & COLLINS, J.L. (1988). Tumor surveillance revisited: natural cytotoxic (NC) activity deters tumorigenesis. *Cell. Immunol.*, **116**, 240.

PETERS, J.H., RUHL, S. & FRIEDRICHS, D. (1987). Veiled accessory cells deduced from monocytes. *Immunobiology*, **176**, 154.

RUSSEL, P.J., PHILIPS, J., ALLAN, W. & 1 other (1988). Antigenic variation and macrophage infiltration of human bladder tumors xenografted into nude mice. *J. Leukocyte Biol.*, **43**, 335.

SIMMONS, D. & SEED, B. (1988). The Fc receptor of natural killer cells is a phospholipid-linked membrane protein. *Nature*, **333**, 568.

STROME, M., CLARK, J.R., FRIED, M.P. & 2 others (1987). T-cell subsets and natural killer cell function with squamous cell carcinoma of the head and neck. *Arch. Otolaryngol. Head Neck Surg.*, **113**, 1090.

VINZENZ, K. & MICKSCHE, M. (1987). Natural cytotoxicity in draining lymphnodes of squamous cell cancer in the maxillofacial region. *J. Oral Maxillofac. Surg.*, **45**, 42.

WHITESIDE, T.L., HEO, D.S., TAGAKI, S. & 1 other (1988). Characterization of novel anti-tumor effector cells in long-term cultures of human tumor-infiltrating lymphocytes. *Transplant. Proc.*, **20**, 347.

WOOD, G.S., WARNER, N.L. & WARNKE, R.A. (1983). Anti Leu3/T4 antibodies react with cells of monocyte/macrophage anf Langerhans lineage. *J. Immunol.*, **131**, 1212.

YAMANAKA, N., HARABUCHI, Y., HIMI, T. & 1 other (1988). Immunosuppressive substance in the sera of head and neck cancer patients. *Cancer*, **62**, 1293.

ZEROMSKI, J., SZEMEJA, Z., REWERS, A. & 1 other (1986). Immunofluorescent assessment of tumor infiltrating cells in laryngeal carcinoma. *Acta Otolaryngol.*, **102**, 325.

ZWADLO, G., BRÖCKER, E.B., BASSEWITZ, D.B. & 2 others (1985). A monoclonal antibody to a differentiation antigen present on mature human macrophages and absent from monocytes. *J. Immunol.*, **134**, 1487.

ZWALDO, G., SCHLEGEL, R. & SORG, C. (1986). A monoclonal antibody to a subset of human monocytes found only in the peripheral blood and inflammatory tissue. *J. Immunol.*, **137**, 512.

ZWADLO, G., VOEGELI, R., SCHULZE-OSTHOFF & 1 other (1987). A monoclonal antibody to a novel differentiation antigen on human macrophages associated with the down-regulatory phase of the inflammatory process. *Exp. Cell Biol.*, **55**, 295.